# A review of the nature and effectiveness of nutrition interventions in adult males – a guide for intervention strategies

**DOI:** 10.1186/1479-5868-10-13

**Published:** 2013-01-29

**Authors:** Pennie J Taylor, Gregory S Kolt, Corneel Vandelanotte, Cristina M Caperchione, W Kerry Mummery, Emma S George, Mohanraj Karunanithi, Manny J Noakes

**Affiliations:** 1 Clinical Research Unit, CSIRO Food and Nutritional Sciences, P.O. Box 10041, Adelaide BC, South Australia 5000, Australia; 2 School of Science and Health, University of Western Sydney, Locked Bag 1797, Penrith, New South Wales 2751, Australia; 3 Centre for Physical Activity Studies, Institute for Health and Social Science Research, Building 18 CQUniversity, Rockhampton, Queensland 4701, Australia; 4 School of Health and Exercise Sciences, The University of British Columbia, 3333 University Way, Kelowna, British Columbia, V1V 1V7, Canada; 5 Faculty of Physical Education and Recreation, University of Alberta, Canada W1-34 van Vliet Centre, Edmonton, Alberta, T6G 2H9, Canada; 6 The Australian e-Health Research Centre, ICT Centre, CSIRO, Level 5, UQ Health Sciences Building 901/16, Royal Brisbane and Women's Hopsital, Herston, Queensland 4029, Australia; 7 CSIRO Food and Nutritional Sciences, P.O. Box 10041, Adelaide BC, South Australia 5000, Australia

**Keywords:** Males, Men, Nutrition, Dietary intervention, Weight loss

## Abstract

**Background:**

Energy excess, low fruit and vegetable intake and other suboptimal dietary habits contribute to an increased poor health and the burden of disease in males. However the best way to engage males into nutrition programs remains unclear. This review provides a critical evaluation of the nature and effectiveness of nutrition interventions that target the adult male population.

**Methods:**

A search for full-text publications was conducted using The Cochrane Library; Web of Science; SCOPUS; MEDLINE and CINAHL. Studies were included if 1) published from January 1990 to August 2011 and 2) male only studies (≥18 years) or 3) where males contributed to >90% of the active cohort. A study must have described, (i) a significant change (p<0.05) over time in an objective measure of body weight, expressed in kilograms (kg) OR Body Mass Index (BMI) OR (ii) at least one significant change (p<0.05) in a dietary intake measure to qualify as effective. To identify emerging patterns within the research a descriptive process was used.

**Results:**

Nine studies were included. Sample sizes ranged from 53 to 5042 male participants, with study durations ranging from 12 weeks to 24 months. Overlap was seen with eight of the nine studies including a weight management component whilst six studies focused on achieving changes in dietary intake patterns relating to modifications of fruit, vegetable, dairy and total fat intakes and three studies primarily focused on achieving weight loss through caloric restriction. Intervention effectiveness was identified for seven of the nine studies. Five studies reported significant positive changes in weight (kg) and/or BMI (kg/m^2^) changes (p≤0.05). Four studies had effective interventions (p<0.05) targeting determinants of dietary intake and dietary behaviours and/or nutritional intake.

Intervention features, which appeared to be associated with better outcomes, include the delivery of quantitative information on diet and the use of self-monitoring and tailored feedback.

**Conclusion:**

Uncertainty remains as to the features of successful nutrition interventions for males due to limited details provided for nutrition intervention protocols, variability in mode of delivery and comparisons between delivery modes as well as content of information provided to participants between studies. This review offers knowledge to guide researchers in making informed decisions on how to best utilise resources in interventions to engage adult males while highlighting the need for improved reporting of intervention protocols.

## Introduction

In terms of dietary behaviours, males are less likely to meet the recommended intakes of fruit and vegetables compared to women [1-5]. According to the *‘Health of Australia’s Males’* report [1] 68% of Australian males are classified as overweight or obese [1,6].

Despite males having a shorter life expectancy [1,7] and being more susceptible to the medical consequences of chronic disease compared to their female counterparts [2,8], participation in preventive health services are lower amongst males [1,2]. Based on current literature, males are less likely to attend face-to-face dietary counselling sessions and tend to be more apprehensive of health-related initiatives, media advertising campaigns, and scientific studies on healthy eating [9]. “Healthy eating” messages are presented by a range of media sources, especially internet-based media, which can provide contradictory nutritional messages to readers or messages based on evidence with unknown scientific quality [2]. For males, who most often do not seek professional consultation [1,2,10], this can lead to self-monitoring of their current health status based on instinctive assumptions from questionable evidence or utilising uninformed partners and friends as a source of advice regarding diet and lifestyle behaviour changes [1,2,10]. A lack of willpower, motivation [11-14] and time [9,15,16] has also been identified by men as the primary barriers that hinder the adoption of healthy dietary behaviours.

Despite research recognising that poor dietary patterns, such as low fruit and vegetable intake and energy excess, as being associated with a greater risk of developing non communicable diseases including diabetes mellitus, heart disease and some cancers [4,6-8], scientific literature specific to lifestyle programs that effectively target engagement by males, however, is very limited. A recent review by Pagoto and collegues [17] noted that only 5% of lifestyle intervention studies targeting weight loss were exclusive to males compared to 32% exclusively targeting females [17]. With recent publications identifying males as a hard-to-reach population and at greater risk of premature death compared to females, interventions should consider appropriate strategies to engage males in improving lifestyle and behaviour outcomes [1,2,8].

Therefore the purpose of this review is to provide a critical evaluation of the nature and effectiveness of nutrition interventions that target the male population. Also to identify strategies that are likely to be effective in improving program engagement by adult males.

## Methods

### Literature search

An individualised search for full-text publications was conducted using the following electronic databases: The Cochrane Library; Web of Science; SCOPUS; MEDLINE and CINAHL. Reference lists of retrieved articles and other relevant systematic reviews [18-20] were also reviewed. The following search terms were used: men OR male; diet OR nutrition OR dietary intake; lifestyle; intervention OR program; fruit and vegetable intake. The search was limited to the adult population (over 18 years of age). Two reviewers independently assessed studies identified in the search for relevance from the title, abstract and key words. Those meeting the inclusion criteria were retrieved and further assessed for relevance. In the event where agreement was not met for studies inclusion/exclusion a third reviewer was engaged.

### Study inclusion criteria

To identify the most effective strategies for improving nutrition and supporting lifestyle risk modification in adult males, this review considers and reports outcomes from studies that have delivered lifestyle interventions aimed at improving dietary intake and/or behaviour in adult males. Nutrition research has grown significantly over time, and more recently to encompass web-based intervention modalities. To ensure relevant current technology and intervention design, dates of publication were restricted to January 1990 to August 2011.

Publications were selected for this review if they met the following criteria:

(i) included adult males only, OR both males and females where data on male participants was reported exclusively AND

(ii) intervention delivered assessed changes in weight and dietary intakes and/or dietary behaviours as a primary or secondary outcome.

Studies were excluded if:

(i) participants recruited had special dietary requirements and dietary interventions targeted these conditions (e.g. diverticular disease; diabetes, heart disease; renal disease; all types of cancers and gastrointestinal disorders) AND

(ii) if studies examined the effect of different diets on weight loss only.

Randomised Control Trials (RCT) with appropriate control group comparisons were the priority for inclusion; quasi-experimental trials (non random allocation) with a comparison group were also considered. If multiple publications presented data from the same population in the same intervention study, only the most relevant publication was included. Although priority was given to nutrition-based interventions, due to the limited body of literature available on nutrition only in males, studies that investigated nutrition in combination with physical activity were also included in the review. Combined studies were excluded in situations where limited data was reported on dietary intake and/or dietary behaviour in combination with physical activity. During the review process a consensus was drawn to include RCT’s where males contributed > 90% of the active cohort and where studies provided substantial insight to nutrition intervention design.

### Data extraction

Data from the included studies, such as methodological quality, intervention and intervention comparison details were independently extracted and quality graded by two reviewers.

Methodological quality of each study reviewed was scored using the McMasters University quality assessment tool for quantitative studies developed by the Effective Public Health Practice Project quality assessment tool [21] . Factors assessed and rated were: selection bias, study design, confounders, blinding, data collection methods and withdrawals/drop-outs. A standardised dictionary developed for the Effective Public Health Practice Project quality assessment tool, was used to classify the factors as strong, moderate or weak (see Table 1).

**Table 1:** Effective Public Health Practice Project Quality Assessment Components and Ratings

**Components**	**Strong**	**Moderate**	**Weak**
Selection Bias	Very Likely to be representative of the target population and greater than 80% participation rate	Somewhat likely to pre representative of the target population and 60–79% participation rate	All other responses or not stated
Design	RCT or CCT	Cohort analytic, case control, cohort or an interrupted time series	All other designs of design not stated
Confounders	Controlled for at least 80% of confounders	Controlled for at least 60–79% of confounders	Confounders not controlled for or not stated
Blinding `	Blinding on outcome assessor and study participants to intervention status and/or research question	Blinding of either outcome assessor or study participants	Outcome assessor and study participants are aware of intervention status and/or research question
Data collection methods	Tools are valid and reliable	Tools are valid but reliability not described	No evidenced of validity or reliability
Withdrawals and dropouts	Follow-up rate of >80% of participants	Follow-up rate of 60–79% of participants	Follow-up rate of <60% of participants or withdrawals and dropouts not described

Individual ratings contributed to a global rating for each study assigning a weak (where 2 or more factors were rated as WEAK); moderate (where less than 4 factors were rated as STRONG and one WEAK rating) and strong (where 4 or more factors were rated as STRONG with NO WEAK ratings). In the event of a discrepancy in interpretation of the findings, all queries were resolved through discussion amongst authors during the review process.

To determine intervention effectiveness, a study must have described, (i) a significant change over the intervention period (p<0.05) in an objective measure of body weight, expressed in kilograms (kg) AND/OR Body Mass Index (BMI) expressed as kilograms per metre squared (Kg/m^2^) in studies with prescribed caloric restriction OR (ii) at least one significant change (p<0.05) in a dietary intake measure including, fruit and vegetable, total energy and total fat intakes.

## Results

### Results of search strategy

Individual data-base searches resulted in 1381 titles and abstracts being identified for this review. Duplicate articles and those that did not meet the specified inclusion criteria, within the title or abstract were removed, leaving a total of 89 publications for consideration. Of these, 18 were review articles and 71 intervention studies. Full text articles were retrieved and assessed against the inclusion criteria. Of the 71 intervention studies, nine met the inclusion criteria for this review (Tables 2 and 3). The majority of the studies excluded from this review included both male and female participants where data was unable to be extracted for males exclusively. All studies targeted overweight and obese males (BMI >25 kg/m^2^) with one study [10] reporting mean baseline BMI <25 kgm^2^. Of the nine studies, eight were RCTs [3,22-28] and one was quasi-experimental [10]. Four (40%) of the included studies were based solely on nutrition interventions in males [3,25,27,28] whilst the remainder combined nutrition and physical activity interventions [10,22-24,26]. All but one study [2] included male participants only. A consensus between the primary and co-authors resulted in this study being included. The basis for its inclusion being that 97% of the participants in the study were male and outcome measures were commensurate with the inclusion criteria.

**Table 2:** Summary of selected intervention studies

**Study**	**Participants**	**Measures**	**Comparisons**		**Duration**	**Outcomes**
			**Intervention/s**	**Comparison**		
Arao et al. (2007) Non-randomised Control trial	· 177 men with risk factors for chronic disease	Primary:	Intervention - LiSM-PAN Group (individual counselling based on stages of change and environmental and social support, work- and home-based)	Control group (Standard Conventional Healthcare (SCH))	6 mths	· LiSM PAN group: showed significant positive changes in leisure time exercise energy expenditure (LEEE) (mean inter-group difference: 400.6 kcal/week, 95% CI: 126.1, 675.0 kcal/week).
Japan	· Wt 68.3 kg (+/- 10.1)	· leisure time exercise energy expenditure (LEEE)	· Individual counselling: 6 month program based on stages of change. 15 mins of one-on-onecounselling on PA and dietary goals. PA goal was specific number of steps/day based on stage of change. Those already active/maintaining given additional PA goals. Structured counselling given by trained professionals	· Generic printed materials on exercise, diet and cooking provided		· No mean inter-group differences reported for dietary habits. (p= 0.432)
	· Ht 166.2 cm (+/- 6.1)	· Secondary:	· Environmental and social support: walking course and exercise facilities installed at workplace, caloric content of lunch menu displayed at workplace café, providing better nutrition through consultation with café manger.	· no counselling nor environmental/social support		· No significant between group difference in changes in dietary fat; fruit and vegetables intakes (0.071-0.238)
	· 40-59 years	· VO_2max_	· Increasing support from family/at home by encouraging participants to discuss health and strategies to improve health with family, men asked to participate in PA with family/spouse, spouse given printed materials on healthy diet/cooking	· Participants given written feedback and recommendations from results of medical check-up and baseline data.		· Greater decreases in BMI, SBP, LDL in intervention vs. control. [<0.001]
		· Intake of fats, fruits and vegetables assessed by FFQ).		Occupational nurse encouraged participants to follow recommendations		· Compliance
		·BMI				· Retention rate for LiSM-PAN group program = 95.2%.
		· BP				· average rate of compliance was 97.1% in monthly counselling
		· Blood glucose				· average achievements of basic target were 86.7% for self-monitoring on the walking steps and 54.7% for controlling dietary targeted activities
		· Lipid parameters				
Booth et al. (2008)	· 54 free-living, overweight or obese males	Primary:	WELL intervention (Weight-loss; exercise; lower blood pressure and longevity intervention group) delivered face to face + 2 2 telephone calls by trained research staff overseen by dietician	Low Fat group (Based on the healthy weight guide by the National Heart Foundation (2002) – no prescribed food volume given) and delivered face to face + 2 telephone calls by trained research staff overseen by dietician	12 wks	Overall:
Randomised controlled trial	· Mean age 48 years	· Changes in dietary intake of: i) fruit, ii) vegetables, iii) dairy)	· Print based material provided on DASH diet with a weight loss focus.	· Generalised written information in the booklet recommended: a) limit high and full-fat foods, b) consume more fruit, vegetables and other plant based products, c) consume fish and legumes at least twice a week.		· 86% retention rate
Australia		· Other measures	· Daily targets set: Participants required to consume at least 4 serves of vegetables, at least 4 serves of fruit, at least 3 serves of dairy and a maximum of 4 serves (4tspn) MUFA.	· Other recommendations: limit high fat foods, choose low fat or reduced fat products, and use a variety of plant based oils for cooking.		· No Difference in mean weight loss between groups 5-6% of TBW lost.
		· BP (taken daily by volunteer)	· Weekly targets set: Participants required to consume 4 serves of nuts and seeds, at least 3 serves of fish, 1 serve of legumes, max 2 serves of red meat.	· Self monitoring through 3-day food diaries completed weekly – each day for 3 consecutive days. Diaries reviewed		· WELL diet achieved a greater Fruit; Vegetable and Dairy intake compared to LG Diet group measured by food group diaries (p<0.01)
		· Weight (taken at each face-to-face visit)	· No restriction on rice/pasta/wholegrain bread and lower-salt cereals as long as they were consuming the volume of other foods listed above.			· (-7.6 7.7 mmHg SBP and -5.4 4.9 mmHg DBP) than LF group (-2.1 6.4 mmHg SBP and
		· Height - baseline	· Self monitoring through 3-day food diaries completed weekly –each day for 3 consecutive days. Diaries reviewed by study staff.			· 1.0 4.1 mmHg DBP (difference in BP change between groups P = 0.001).
		· BMI				
Braekman et al (1999)	· 638 middle aged men	Primary:	Low-Fat Dietary Intervention	Control sites (no access to support)	3 mths	· 82% retention rate at 3 months
Randomised controlled trial (4 worksites randomised)	· Mean age 43.7 (+/-6.6)	· Dietary Habits (24 hr food record)	· Participants informed of baseline screening measures at 2 weeks through individual counselling session and informed of personal risk factor profiles	· Provided written summary of risk factor profile with nil dietary education/information provided. Exception for those with abnormal values who were referred to their GP.		· Significant reduction in total energy and total fat intake in the intervention group (p<0.05) but no difference for percent of energy from types of fat.
Belgium	· Mean BMI 26.5 kg/m^2^	· Serum Lipid levels	· Mass media used within Intervention sites to stress the link between Cholesterol and heart disease and the role of a low fat diet.			· Intervention group increased protein and carbohydrate more than the control (p<0.05)
		· Secondary:	· Poster displays and leaflets providing strategies on how to reduce dietary fat provided at intervention sites			· BMI increased by 0.3 kg/m^2^ in the intervention group vs. controls (p<0.001)
		· (Self administered Health Questionnaire (smoking; PAL and medical history)	· Video outlining importance of reducing blood cholesterol by reducing dietary fat intake presented with question and answer time at a worksite safety meeting			· Nutrition knowledge significantly greater in the intervention groups (p<0.001) No significant effect for total cholesterol between groups
		· Nutrition knowledge (10-item questionnaire)	· Participants offered several non-compulsory dietician-led 2 hour dietary group education sessions at the worksite out of work hours			· HDL cholesterol increased in the control group compared to intervention group (p<0.001)
		· WHR	· Summary newsletter provided at the end of the study to reinforce dietary messages			
		· BMI				
Leslie et al. (2002)	· 122 overweight/ obese males	Primary	Energy Deficit diet (ED) (a 2512 kJ (600 kcal deficit)with individualized energy prescriptions	Generalised low calorie diet (6279 kJ=1500 kcal)	24 wks	· Weight loss significant in both ED and GLC groups but no difference between groups in weight loss or maintenance.
					(12 wks intervention + 12 wk maintenance)	
Randomised controlled trial	· 18-55 years	· Weight loss	1. ED with meat	3. GLC (general low calorie) meat		· No effect of meat vs no meat on weight loss or biochemical measure between groups
United Kingdom		· Weight loss maintenance	2. ED no meat	4. GLC no meat		· Significantly more attrition from the GLC group than the ED group.
		· Secondary	All attended initial dietary consult (60 minute) delivered by dietician and face-to-face reviews every 2 weeks for 20 minutes for first 12 weeks.			· 69% Retention at 24 weeks.
			All groups underwent 12 weeks weight loss followed by 12 week maintenance phase			
			All contacted by email at 2 week intervals and self reported anthropometric and dietary information requested.			
		· Lipids (plasma)				
		· Dietary Habits				

Note: BMI= Body Mass Index; WC = Waist Circumference; BP = blood pressure, FFQ = Food Frequency Questionnaire, LEEE = leisure time exercise energy expenditure; WHR = Waist to hip ratio.

**Table 3:** Features of study interventions by setting, personnel, mode of delivery and form of nutrition information

**Study**	**Setting**	**Length of intervention**	**Personnel administering delivery**			**Mode of delivery**					**Form of Nutrition Information**	
		**Short-term (<6 mths) Long-term (>6 mths)**										
			**Researcher**	**Dietician**	**Exercise Physiologist**	**Face-to-Face**	**Group**	**Phone**	**Internet**	**Print**	**Prescriptive / Tailored**	**Generic**
Arao et.al (2007)	Worksite	Short-term	√			√				√	√	√
			Not defined									
Japan												
Booth et al. (2008)	Community	Short-term	√	√		√		√			√	√
			not defined	not defined								
Australia												
Braeckman et al. (1999)	Worksite	Short-term		√		√	√			√		√
				not defined								
Belgium												
Leslie et al. (2002)	Worksite	Short-term		√		√			√		√	
United Kingdom				not defined								
Morgan et.al (2011b)	Worksite	Short-term	√			√			√	√		√
			Male									
Australia (POWER)												
Morgan et al. (2009, 2011c),	Community (Family)	Short-term	√			√				√		√
Collins et al 2011												
			Male									
Australia (SHED-IT)												
Morgan et al. (2011a)	Community (Family)	Short-term	√			√	√		√	√		√
			Male									

Australia (HDHK)												
Pritchard et al. (1997	Worksite	Long-term		√	√	√				√	√	√
				not defined	not defined							
Australia												
Tilley et al. (1999, 1997)	Worksite	Long-term	√			√	√	√		√		√
			Not defined									
USA												

### Description of included studies and participants

A total of 6,167 overweight/obese males were represented in these nine studies. Sample sizes of the included studies ranged from 53 to 5,042 male participants and were aged 18 years and over. Studies were based in Australia [3,18,23,24,26], Belgium [27], Japan [10], United Kingdom [25] and the United States of America [28]. Six studies were worksite interventions [10,24-28] and three were community-based interventions [3,22,23] of which two involved family members [22,23]. Study duration ranged from 12 weeks to 24 months, of these seven were considered short-term (<6 months) [3,10,22-25,27] and two long-term (>6 months) [26,28].

Eight studies observed weight changes [3,10,22-27], three studies [22,25,26] had the primary aim of achieving weight loss through caloric restriction whilst five studies [3,10,24,26-28] focused on achieving changes in dietary intake patterns relating to modifications in fruit, vegetable, dairy and total fat intakes. Four studies reported the social cognitive theory as supporting the intervention [22-24,28].

The characteristics of each study including participants, the intervention, comparison, duration and outcomes that reported changes in dietary behaviours and/or weight changes were stratified. Additional features of the intervention were further tabulated by personnel delivering the intervention (e.g. dietician), mode of delivery (e.g. print, internet or face-face) and form of nutrition information provided (e.g. generic or tailored).

### Personnel

Interventions were dietician-led [3,25,27] dietician and exercise physiologist-led (sex not defined) [26] male researcher-led [22-24] or researcher-led (sex non-defined) [10,29].

### Quality of included studies

Assessment of study quality identified four studies as having a strong quality rating [10,22,24,26], four rated as moderate [3,23,25,28] and one rated as weak [27]. Quality Assessment results can be seen in Table 4.

**Table 4:** Quality assessment results against the Effective Public Health Practice Project Quality Assessment Tool for all included studies

**Author/Date**	**Selection Bias**	**Design**	**Confounders**	**Blinding**	**Data Collection Methods**	**Withdrawals/ Dropouts**	**Global ratings**
Arao et al. (2007)	Moderate	Strong	Strong	Moderate	Strong	Strong	**Strong**
Booth et al. (2008)	Moderate	Strong	Moderate	Moderate	Strong	Moderate	**Moderate**
Braekman et al. (1999)	Weak	Moderate	Moderate	Weak	Moderate	Moderate	**Weak**
Leslie et al. (2002)	Strong	Strong	Moderate	Strong	Moderate	Moderate	**Moderate**
Morgan (2009, 2011c) SHED-IT	Moderate	Strong	Strong	Moderate	Strong	strong	**Strong**
Morgan et al. (2011a) HDHK	Moderate	Strong	Moderate	Weak	Strong	strong	**Moderate**
Morgan et al. (2011b) POWER	Moderate	Strong	Moderate	Moderate	Strong	Strong	**Moderate**
Pritchard et al. (1997)	Strong	Strong	Moderate	Moderate	Strong	Strong	**Strong**
Tilley et al. (1999, 1997)	Strong	Strong	Moderate	Weak	Moderate	Moderate	**Moderate**

Those that were rated moderate and weak performed poorly in the quality assessment criteria for selection bias, confounders and data collection methods. Study designs differed substantially in the nature of the interventions and the nature of the control group. For example, some studies compared the intervention to a wait-listed, no intervention control group [23,24,27,28] whilst others compared two different forms of intervention [3,10,22,25,26]. Several modes of delivery within studies were common such as combining print-based materials (n=8) [10,22-24,26-29], face-to-face (n=9) [3,10,22-29], or phone counselling (n=2) [3,28] and use of internet-based tools (n=3) [23-25].

### Intervention effectiveness

Intervention ‘effectiveness’, as defined previously, was found for seven of the nine studies and is shown in Table 5. Five effective studies were in the worksite setting [10,24,26-28] and two were community based [3,23]. One community study focussed on overweight males and their primary school aged children [23]. Four studies reported using a theoretical basis for the intervention utilising social cognitive theories and readiness to change models [10,23,24,28]. The duration of study interventions were short-term, 12 weeks to six-months [3,23,24,27] and long-term 12–24 months [26,28]. Interventions targeted nutritional intake and dietary behaviours [3,27,28] and dietary intake in combination with physical activity [10,23-25]. Pritchard et al. was the only study that compared weight loss through caloric restriction against weight loss through exercise only to a control group whereby participants attended weight monitoring sessions only [26].

**Table 5:** Dietary evaluation summary of interventions to improve Men’s nutritional and weight-loss outcomes - Effectiveness table

**Study**	**Weight status/lipid studies/BP**		**Nutritional determinants**		**Nutrition Intake**		**Intervention Effectiveness**
Arao et al. (2007)	BMI	✓	Dietary control	×	Fat intake (serves)	×	YES
	Lipid studies	✓			Fruit intake (serves)	×	
	Blood Pressure(mmHg)	✓			Vegetable intake (serves)	×	
	Blood glucose	✓					
Booth et al. (2008)	Body Weight (kg)	×	-		Fat intake (serves)	✓	YES
	BMI	×			Fruit intake (serves)	✓	
	Blood Pressure(mmHg)	✓			Dairy intake (serves)	✓	
Braekman et al. (1999)	BMI	×	Nutrition knowledge	✓	Total Energy and macronutrient intake	✓	YES
	Lipid studies (HDL)	✓					
Leslie et al. (2002)	Height (cm)	×	Dietary practices monitored through dietary	×	Total energy intake	×	NO
	Body Weight (kg)	×	targets monitor				
	Waist Circumference (cm)	×					
	Lipid studies	×					
Morgan (2009, 2011c) SHED-IT	Body Weight (kg)	×	Dietary knowledge and belief cognitions	×	Total energy intake	×	NO
	BMI	×	Quality of Life & general health measures	×			
	WC (CM)	×	Frequency of take-away food consumption	×			
	BP (mmHg)	×	& eating while watching TV				
Morgan et al. (2011a) HDHK	[fathers] Weight (kg)	✓	Social support	na	Total energy intake	×	YES
	BMI	✓	Intentions				
	WC	✓	Self-efficacy				
	BP	✓	Outcome expectations				
Morgan et al. (2011b) POWER	Body Weight (kg)	✓	Dietary patterns	✓	Dietary behaviours - Sweetened beverages (Serves).	✓	YES
	BMI	✓	Dietary cognitions.	✓			
	WC	✓					
	BP	✓					
Pritchard et al. (1997)	Body Weight (kg)	✓	-		Energy intake (kcal)	×	YES
	Fat Mass	✓			Percentage dietary fat	×	
Tilley et al. (1999, 1997)	-		-		Total Fat (% Energy)	✓	YES
					Fibre intake (g/1000kj)	✓	
					Fruit & Vegetable intakes (Serves)	✓	

Note: ✓ = Significant between group difference identified (See table one) ; × = NO statistically significant change between intervention vs control identified.

### Effectiveness of interventions aiming to achieve weight loss

Of the effective interventions, four studies [10,23,24,26] reported significant positive changes in weight (kg) and/or BMI (kg/m^2^) changes (p ≤0.05). Study sample sizes ranged from 53 to 177.

Two studies involved internet usage [23,24] including the use of a commercially available self-monitoring tool in combination with face-to-face group interaction [23,24] and an independent study website [24]. All other studies provided face-to-face, individual intervention delivery [10,23,26]. All studies used combinations of nutrition and physical activity strategies to promote weight-loss [10,23,24,26] and two used caloric restriction through personalised low-fat dietary plans aimed at reducing baseline intake by 500kcal per day [23,26]. Other nutrition strategies included reduction of total fat intake, portion serves and building awareness of targets for healthier eating practices [10,23,24,26].

### Effectiveness of interventions aiming to achieve change in dietary intake and food behaviours

There were four effective interventions (p <0.05) targeting determinants of dietary intake and dietary behaviours and/or nutritional intake [3,24,27,28]. Study sample sizes ranged from 53 to 5042. One study included single face-to-face interaction, use of a commercially available self-monitoring tool and access as needed to an independent study website [23]. Other modes of delivery included multiple face-to-face interactions combined with phone support [3] or non-compulsory group education sessions [27]. Tilley et al. conducted a long-term study, duration of 2 years, incorporating multiple group face-to-face interactions with phone support [28]. A range of dietary behaviours were targeted, most commonly a combination of total fat reduction and increasing daily fruit and vegetable intake [3,27,28], dietary patterns and/or nutrition knowledge [3,24,27].

### Mode of intervention delivery

#### Group-based delivery

Three group-based intervention studies were included in this review [23,27,28]. All three delivered face-to-face group sessions and print-based materials in a group-based setting to convey information on healthy eating [27,28] and physical activity [23]. One of these studies included telephone delivery of motivational tips and feedback on diet quality during the intervention [28]. Two studies were worksite interventions [27,28] and one study accessed families in the community [23]. Interventions were dietician-led (sex non-defined) [27], male researcher-led [23] or researcher-led (sex non-defined) [28]. The “Healthy Dads, Healthy Kids” study included the use of a publically available website, CalorieKing™, for self-monitoring dietary intake and physical activity habits. Fifty-three obese males (mean age 40.6 +/− 7.1 years) and their primary school aged children (n=71) participated in the study which compared a structured, 8 week face-to-face group program known as the “Healthy Dads, Healthy Kids” program, against a wait-list control group [23]. The program followed the constructs of Social Cognitive Theory and Family Systems Theory and delivered dietary information based on the Australian Guide to Healthy Eating’s recommended daily intakes, meal planning, food label reading and goal setting in addition to physical activity messages [23]. There was a significant between-group difference in weight loss at 6 months (7.6 kg vs 0.0 kg, P<0.001 for treatment effect), and a significant correlation was identified between percentage weight loss and number of diet entries, daily physical activity entries and weekly weight check-ins. There was no measurable effect for changes in total energy and dietary intake [23]. The authors noted that targeting fathers for lifestyle interventions improved the health behaviours of their children [23].

The worksite interventions included greater numbers of participants (n=638) [27], (n=5,042) [28] compared to the community run intervention (n=53) [23]. The study by Braeckman and colleagues [27] recruited 638 males (mean age 43.7 +/− 6.6) from 4 worksites to participate in the study comparing a 3-month generic low-fat dietary intervention with control group sites, where no dietary education or information was provided [27]. The intervention sites were exposed to a range of media, including posters, leaflets, newsletters and videos outlining the importance of a low-fat diet in reducing the risk of heart disease and provided optional 2-hour dietician-led dietary group education sessions which were run out of business hours [27]. At 3 months there was a significant between-group difference for total energy and total fat, with the intervention groups reporting a reduction of 8.6% and 7.2% respectively (p<0.05) and a significant improvement of 44.5% in nutrition knowledge (p<0.001) [28]. Body mass index (BMI) for the intervention group increased by 0.3 kg/m^2^ compared to the control group and the authors suggest an increase in BMI was observed in the intervention group as a result of participants overeating “healthier” foods and under-reporting post intervention dietary intakes [27].^.^ There was no measurable effect for percent of energy from types of fat or total cholesterol between groups [27].

The final study by Tilley and colleagues [28] recruited 5042 participants from 28 male dominant worksites (mean age 56.5 +/− 12.2 years) into the Next Step Trial; >90% of this cohort were males [28,29].

The Next Step Trial was a multi-component, cancer control program, which compared a generic 2-year face-to-face group program against control groups [28,29]. The Next Step Trial followed the constructs of Social Cognitive Theory and the transtheoretical model of change to deliver dietary information that promoted a reduced fat dietary intake and an increased fruit and vegetable intake according to the USDA food guide pyramid [28,29]. Five nutrition education groups, provided during paid working hours, delivered information regarding diet quality, behaviour change, goal setting, tips on how to obtain family and co-workers support and information regarding the importance of cancer screening [28,29]. One motivational telephone call was delivered to participants, and group attendance records were maintained [28,29]. Computer generated personalised feedback was provided to participants in the form of graphs comparing employee’s dietary intake against the USDA food guide pyramid [28,29]. The intervention sites were exposed to a range of media including posters, leaflets and newsletters on how to reduce dietary fat and increase fruit and vegetable intake [28,29]. There was a significant between-group effect for all outcome measures at 12-months (p<0.006), with total energy reducing by 1.5%, dietary fibre increasing by 0.5 g/1000Kcal and daily fruit and vegetable servings increasing by 0.13 per day, with a significant effect observed for fibre only at 24-months (p =0.002) [28,29]. A significant dose–response was observed for group attendance rates, with those attending all 5 group sessions (19%) reducing their total energy from dietary fat by 3%, increasing dietary fibre by 1 g/1000Kcal and fruit and vegetable intakes increasing between 0.3 to 0.7 serves per day [28,29].

This study identifies that participation in worksite health promotion initiatives where employers enable employees paid release time from work to attend education sessions may be an effective strategy to enhance intervention effectiveness [28,29].

#### Face-to-face delivery

Face-to-face methods featured in all nine interventions [3,10,22-28]. All studies combined face-to-face delivery with at least one of the following additional modes of delivery including internet [3,22-25] or print-based material [10,22-24,26-28], telephone contact [3,28] and group-based contact [23,27,28]. Participants from six of the studies were recruited from worksite environments [10,24-28] whilst three studies recruited from the general community [3,22,23]. The frequency of face-to-face contact varied between one session only [19]; fortnightly [3,22,24-27] and monthly [10,28]; with nutrition education sessions led by a dietician [3,25-27], male researcher [22-24], or researcher (sex not defined) [10,28]; with time for dietary intervention delivery ranging from 15 minutes to two hours [3,10,22-28]. Of the nine studies, four had developed the behaviour change intervention based on Social Cognitive Theory [10,22,23,28] to guide goal setting and self-monitoring behaviours and to guide researcher feedback on participant performance [10,22,23,28]. All face-to-face studies included the provision of individualised feedback on dietary intake patterns, adherence to targets and weight loss goals to participants. Of the nine studies, only three reported significant positive between-group effects for changes in dietary intake patterns [3,27,28]. The comparison groups in these studies were generic low-fat dietary intervention [3], no access to information or support [27,28].

Booth and colleagues [3] studied the impact of prescribed dietary targets vs. generalised information for fruit, vegetable, and dairy consumption in a cohort of 63 males (mean age 48 years) over a 12-week period [3]. Dietician-led face-to-face sessions were conducted fortnightly and supplemented with two phone counselling sessions over the study duration [3]. Findings by Booth et al. suggested that the setting of quantitative-based dietary targets performed better than generic information in improving self-reported dietary behaviours [3].

#### Internet-based delivery

Three studies reported utilising internet technology; three were work-site based programs utilising on-site intranet access including email [24,25] and one was a community based program [23]. The common internet-based features utilised in the abovementioned studies were: interactivity with the user [22-24]; personalised feedback based on user entered food data [23-25]; and use of transtheoretical behaviour change constructs within nutrition messages [22-24]. The Transtheoretical model assesses an individual's readiness to act on a new healthier behaviour and provides strategies or processes of change to guide the individual through the stages of change to action and maintenance [30].

Leslie et al. conducted a study where a dietician delivered generic dietary information via email in combination with face-to-face sessions [25]. Although weight loss was significant in both intervention groups compared to the control group, adherence with dietary advice was limited [25].

## Discussion

This review evaluated recent literature where interventions targeted males in community and worksite settings as a means to improve nutrition and dietary behaviours. From the nine studies included in this review, seven were considered effective in achieving satisfactory outcomes [3,10,22-24,26-28]. Few male-based dietary intervention studies have focussed primarily on weight management [10,22-24,26], and to a lesser extent on diet quality changes [3,25,27,28]. Many lacked sufficient detail on intervention description and few described any behavioural theory underpinning the approach used. This makes interpretation of the effectiveness of different delivery modes difficult to determine, with this review suggesting that intervention effectiveness was not associated with mode of intervention delivery.

Little information exists for why males are under-represented in dietary modification interventions [17], suggesting that future investigations need to consider the barriers preventing males from engaging in dietary modification strategies. This was reinforced by Robertson et al. who were unable to determine any effective approaches to enhance the uptake of health services by males [18].

This review identified an increasing trend towards recruitment of male participants within the workplace. Six of the nine studies included in this review recruited male participants from worksite environments [10,24-28], two incorporated an internet-based methodology for delivering interventions [24,25]. This may suggest that utilising worksites where the internet is readily available to employees may be an effective delivery mechanism with the potential to be a wide reaching and cost-effective option for targeting the male population [18-20,31].

An important issue for interventions is getting participants to effectively and frequently engage in the provided programs. Previous studies have shown that higher use of internet-based features combined with telephone interaction proved to be effective in changing dietary behaviours [19,32]. The features of successful internet-based lifestyle change programs remain to be identified, and as noted by Neve and colleagues [19], it is not yet possible to attribute the effectiveness of internet-based interventions to specific intervention components due to heterogeneity of study designs. Manzoni et al. supported this view but noted greater efficacy of behavioural internet-based programs which included tailored feedback on self-monitoring of weight, eating and activity over education only internet-based interventions [33]. Qualitative feedback received through semi-structured interviews from participants of the SHED-IT trial strengthen the comments made by Manzoni et al. reporting that most respondents found that Calorie King™ was a invaluable component of the SHED-IT program, reported to aid in their understanding of the effects of diet and exercise on weight loss due to the instant and visual feedback gained online [34]. However, limitations for the use of Calorie king™ existed including time needed to enter daily intake, navigation of the website was not intuitive and respondents suggesting that the foods contained within Calorie King™ did not cater for those who did not dependant of fast foods, pre-prepared or standard foods [34].

To enhance effectiveness of dietary interventions for males, literature suggests that nutrition messages need to remain clear, concise and achievable [3,35]. Messages are also well received when they are based on the participants’ identified needs and presented frequently in an engaging and fun manner [3,10,23,28,35]. This result is consistent with Morgan et al.’s evaluation of the SHED-IT intervention, which highlighted that males were attracted to programmes that do not require extensive time commitment but present key nutrition messages in a thoughtful but comical manner [34]. Practical advice, such as simple weekly meal plans that are inclusive of “treat” foods and beverages recipes, and the engagement of wives or partners, has been deemed helpful to overcome the barriers to healthy eating in males [14,34].

Several studies demonstrated the importance of self-monitoring (e.g. keeping dietary intake records, tracking/monitoring weight online) to enhance adherence and encourage long-term behaviour change [3,22-25,28]. Self-monitoring is strongly associated with program adherence and positive long-term behaviour change. Previous studies have recognised that more frequent self-monitoring of dietary behaviour is positively correlated with weight loss independent of delivery mode [36-39].

Although excluded from this review due to the lack of any control group comparison, Auon et al. provide valuable insight on how collaboration between health care service providers and existing community groups/clubs can lead to sustainable lifestyle strategies for males [40].

Auon et al. [40] delivered a community-based lifestyle intervention program recruiting 750 overweight males from 23 Rotary Groups. The program was titled the “Waist Disposal Challenge” and was delivered by health service dieticians and exercise physiologists. These included face-to-face group educational presentations (up to three over 12 months), a face-to-face BMI monitoring/competition - weight and height measured and recorded monthly with competition between Rotary clubs and telephone lifestyle coaching through four calls to each participant [40]. This study found significant decreases in BMI; increased awareness of lifestyle changes that promote weight loss; and increased motivation through friendly club competition, suggesting that face-to-face group presentations on nutrition and physical activity may help males to facilitate lifestyle changes [40]. This perception was also observed by Morgan et al.’s findings whereby participants of the SHED-IT trial suggested improvements for future trials be to consider increasing face-to-face contact in the form of small groups and meetings [34].

Also highlighted was that similar community-based projects that utilise existing community groups/clubs in collaboration with health care service providers, potentially enable interventions to be self-managed and sustainable [40]. The study undertaken by Morgan et al. [24] with male shift workers clearly demonstrates that information sessions, program booklets, group-based financial incentives and an online component in the Workplace Power Program was able to achieve reductions in weight and soft drink consumption [41]. However, Collins et al. [42] noted that whilst males were able to reduce portion size in the SHED-IT Program, reductions in alcohol or increases in fruit and vegetable intake were not achieved, recommending that specific food based guidelines be included in future programs for males [41,42].

### Strengths and limitations

The strengths of this review are that it draws on evidence from randomised control trials targeting males and systematically assesses the methodological quality of studies. However, it is not without limitations. Although the majority of the studies in our review reported effective interventions, the findings from this review are based on a small number of studies of which trials were limited by short intervention periods and absence of long-term follow-up. Additionally publication bias could be a result of the broad search focus that may have resulted in relevant papers being missed; this is in addition to unpublished (grey literature) not being sought.

## Conclusion

The area of male health has been recently ignited on the health agenda [1,2] but remains under-developed in exploring effective dietary interventions. Although seven of the nine reviewed studies were effective in achieving weight-loss outcomes and/or change in dietary practices in the short-term, uncertainty remains as to the primary features of successful dietary interventions for males due to limited details provided for nutrition intervention protocols, variability in mode of delivery and comparisons as well as content of information provided to participants between studies. However, the reviewed studies do provide valuable insight to potential recruitment and intervention strategies for this hard to reach male population, favouring worksite recruitment practices where employer engagement and intervention support is obtained.

To enable evaluation of intervention effectiveness between studies it is recommended that larger scale effectiveness studies that report nutrition intervention protocols and content of information provided in males (or gender stratified) are needed.

Although outside the scope of this review, studies exploring the cost-effectiveness of worksite vs community interventions targeting eating behaviour and energy balance would be valuable to explore.

## Competing interests

Corneel Vandelanotte is supported by a National Health and Medical Research Council of Australia (#519778) and National Heart Foundation of Australia (#PH 07B 3303) post-doctoral research fellowship.

All Authors declare that they have no competing interests that are directly relevant to the content of this review.

## Authors' contributions

PJT contributed to the literature review, prepared initial draft of the manuscript, rated and tabulated primary articles and prepared final draft for publication. GSK contributed to the conceptual phase and editing of the manuscript and drafts. CV contributed in the conceptual phase of the paper as well as editing drafts of manuscript. CMC contributed to conceptual phase and editing of the manuscript and drafts. KWM contributed to the conceptual phase and editing of the manuscript and drafts. ESG contributed to the literature review and in the editing of the manuscript. MK contributed to the conceptual phase and editing of the manuscript and drafts. MJN contributed to the project development, preparation and editing of the manuscript and ranking and tabulation of primary articles. All authors read and approved the final manuscript.
